# The Smoke and Fog of London

**Published:** 1881-07

**Authors:** 


### POPULAR SCIENCE DEPARTMENT.

### THE SMOKE AND FOG OF LONDON.

It has long been known that the atmosphere of London is seriously impaired at all times, by the smoke proceeding from the use of bituminous coal as the nearly exclusive fuel of that city, and that the greatest source of complaint from those "London fogs" which proceed from certain geographical conditions, pertaining to the British Isles, and to the locality of London in particular, is found in the same smoke. "The prevention of smoke" has consequently been an open subject for discussion for the past two centuries, as a matter of printed record, and possibly for much longer time in fact.

The introduction of the use of coal gas for lighting, has brought into limited use, so far in London, a non-smoke producing fuel, coke, which, if generally burned, would subserve the same end in affording relative purity to the air that the use of anthracite coal does to *some* American cities. The use of coke for the domestic fuel of a city is not without example both in effect and result ; for the city of Paris is thus supplied at the present time, with the advantage of a clear atmosphere and of clean houses and personalities. The dinginess of English cities is almost indescribable, although Pittsburgh, Wheeling or Cleveland approach the dismal stand of high and low life in a chimney flue.

It is now proposed by Mr. W. D. Scott-Moncrieff, C. E. (in a lecture before the Society of Arts, London), that in the process of making coal gas, much less gas shall be removed from the coal, thus producing a superior article of coke—coke practically "20 per cent. superior," in its heat producing qualities for domestic use, to the original coal itself. Gas extracted in this way will be better for illumination than the present coal gas, for with such coal gas a burner consuming five cubic feet of gas per hour will evolve the light of 24 candles in place of 16 candles, as now. The average amount of gas extracted from a ton of coal (2,240 lbs.) is 10,000 cubic feet. The lecturer had proved as the result of practical experiments, that by extracting only 3,000 to 5,000 cubic feet per ton, there remained an excellent and smokeless coke.

The inference to be drawn from the report of the lecture before us, is that at present 2,000,000 tons of coal are treated annually in London for the production of coal gas, while the total demand for consumption is but 6,000,000 tons. By enlargement of the gas works it is proposed that the entire quantity of coal be treated with the extraction of 3,333 cubic feet of 24 candle gas from each ton. Upon these bases, the citizens of London will derive 20 per cent. more heat from the fuel for all the purposes of heat demand, domestic or manufacturing; the gas works will derive the advantages of a better article of gas for giving out light, and also a further advantage in profits derived from by-products of gas-making, which will have been largely increased.

At the conclusion of the lecture, it was said "statistics showed that if the system were carried out, there would be an annual saving of £2,125,000, which might be taken as the yearly value of London smoke. London would then be a smokeless city, and all that would have to be deducted from the £2,125,000 per annum would be confined to a few items, such as the cost of additional capital required for transit, appliances, and the terms to be made with the gas companies for carrying out the scheme."

